# Increased Carotid Siphon Tortuosity Is a Risk Factor for Paraclinoid Aneurysms

**DOI:** 10.3389/fneur.2022.869459

**Published:** 2022-05-10

**Authors:** Shilin Liu, Yu Jin, Xukou Wang, Yang Zhang, Luwei Jiang, Guanqing Li, Xi Zhao, Tao Jiang

**Affiliations:** ^1^Department of Neurosurgery, The Second Affiliated Hospital of Anhui Medical University, Hefei, China; ^2^Department of Neurology, Bozhou City Peoples Hospital, Bozhou, China; ^3^Department of Neurosurgery, The First Affiliated Hospital of Anhui Medical University, Hefei, China; ^4^Philips Healthcare China, Shanghai, China; ^5^Anhui Provincial Institute of Translational Medicine, Hefei, China

**Keywords:** morphology, internal carotid artery, carotid siphon, paraclinoid aneurysm, risk factor

## Abstract

**Background:**

Geometrical factors associated with the surrounding vasculature can affect the risk of aneurysm formation. The aim of this study was to determine the association between carotid siphon curvature and the formation and development of paraclinoid aneurysms of the internal carotid artery.

**Methods:**

Digital subtraction angiography (DSA) data from 42 patients with paraclinoid aneurysms (31 with non-aneurysmal contralateral sides) and 42 age- and gender-matched healthy controls were analyzed, retrospectively. Morphological characteristics of the carotid siphon [the posterior angle (α), anterior angle (β), and Clinoid@Ophthalmic angle (γ)] were explored *via* three-dimensional rotational angiography (3D RA) multiplanar reconstruction. The association between carotid siphon morphology and the formation of paraclinoid aneurysms was assessed through univariate analysis. After this, logistic regression analysis was performed to identify independent risk factors for aneurysms.

**Results:**

Significantly smaller α, β, and γ angles were reported in the aneurysmal carotid siphon group when compared with the non-aneurysmal contralateral healthy controls. The β angle was best for discriminating between aneurysmal and non-aneurysmal carotid siphons, with an optimal threshold of 18.25°. By adjusting for hypertension, smoking habit, hyperlipidemia, and diabetes mellitus, logistic regression analysis demonstrated an independent association between the carotid siphons angles α [odds ratio (OR) 0.953; *P* < 0.05], β (OR 0.690; *P* < 0.001), and γ (OR 0.958; *P* < 0.01) with the risk of paraclinoid aneurysms.

**Conclusions:**

The present findings provide evidence for the importance of morphological carotid siphon variations and the likelihood of paraclinoid aneurysms. These practical morphological parameters specific to paraclinoid aneurysms are easy to assess and may aid in risk assessment in these patients.

## Introduction

The mechanism of intracranial aneurysm formation may be the result of congenital defects of the arterial wall and a variety of other acquired factors (1). Experimental and clinical evidence suggests a role for hemodynamics in aneurysm initiation, growth, and eventual rupture (2). The morpphology of blood vessels represents a major determinant of hemodynamic patterns and can present a more practical model for understanding complex hemodynamics (3). Most studies have focused on complicated three-dimensional morphological features of the aneurysmal sacs and parent vessels, such as undulation indices, inflow angles, and non-sphericity indices (4–6). Arterial bifurcations and high-vascular curvature indicate a higher risk for aneurysm formation (7) and the carotid siphon is the primary flexible part of the carotid artery and is one of the sites highly susceptible to vascular lesions (8). In this study, the morphology of the carotid siphon will be discussed, and its importance is highlighted as a natural tortuous vessel segment with sharp bends, giving rise to the anterior brain circulation (9).

The internal carotid artery (ICA) is a segment of the artery where aneurysms commonly initiate and accounts for nearly 30% of all intracranial aneurysms (10). Paraclinoid aneurysms are defined as those arising from the segment of the ICA between the roof of the cavernous sinus and the origin of the posterior communicating artery (PcoA) and account for approximately 5–14% of all intracranial aneurysms (11, 12). Because of the complex anatomical relationship between neurovascular, dural, and bony structures, the presence of paraclinoid aneurysms associated with the morphology of the ICA carotid siphon warrants further investigation when comparing other intracranial aneurysms (13). Correlations between vascular geometry and the occurrence of paraclinoid aneurysms have been reported, but, as with hemodynamic studies, the morphologic results have not always been consistent (10, 14–18). Given, the clinical practicality and the controversial findings seen with complicated morphometric measurements, we selected basic geometrical parameters of carotid siphon as independent variables.

The aims of this study, therefore, were to evaluate the morphological characteristics of the carotid siphon associated with and without an aneurysm. And to look for a potential link between carotid artery tortuosity and spontaneous paraclinoid aneurysm formation, development, and incidence.

## Methods

### Patient Selection and Data Acquisition

**Figure 1:**
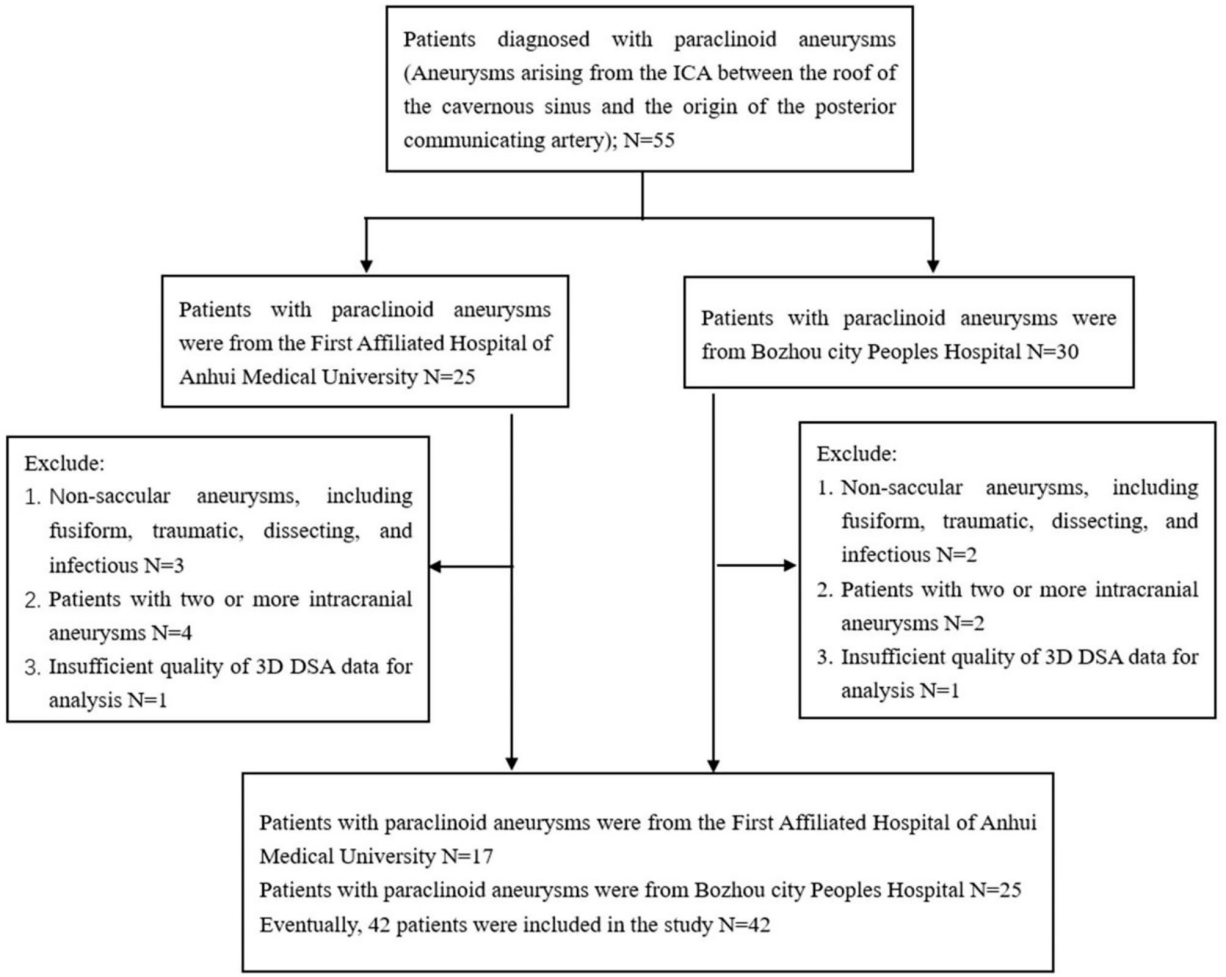
The flow chart of paraclinoid aneurysms participant enrollment.

Imaging data and medical records were retrospectively reviewed from 55 patients with paraclinoid aneurysms who were admitted to the First affiliated hospital of Anhui Medical University and Bozhou City Peoples Hospital, between September 2018 and June 2021. This study was approved by our Institutional Review Board and the requirement for informed consent was waived by the committee due to the retrospective nature of the study. Inclusion criteria were: aneurysms arising from the ICA between the roof of the cavernous sinus and the origin of the posterior communicating artery. Exclusion criteria were: (a) non-saccular aneurysms, including fusiform, traumatic, dissecting, and infectious; (b) two or more intracranial aneurysms; (c) insufficient quality of the 3D DSA data to enable analysis. Inclusion and exclusion criteria are reported in Figure 1. A total of 42 patients with 42 paraclinoid aneurysms were included in the study, 31 of whom had unilateral paraclinoid aneurysms with imaging data for the unaffected contralateral side. Therefore, a matched case-control analysis was included.

In total, 42 patients with paraclinoid aneurysms were divided into two subgroups according to different anatomical locations of the internal carotid artery (19) as follows: 17 located on the C5 (clinoid segment) and 25 located on the C6 (ophthalmic segment).

Furthermore, 42 age- and sex-matched healthy patients with no aneurysms selected from the database served as controls. Therefore, the aneurysm and control groups were matched by the demographic criteria only. Each patient underwent digital subtraction angiography (DSA) and three-dimensional (3D) reconstructions (Philips Medical Systems, Best, The Netherlands) to characterize the anatomy of the parent artery and aneurysm. All the aneurysm geometries were measured by DSA and demographic information and clinical data, including patient age, sex, tobacco use, hypertension, hyperlipidemia, and diabetes mellitus were retrieved from the medical records.

### Analysis of Morphological Parameters

**Figure 2:**
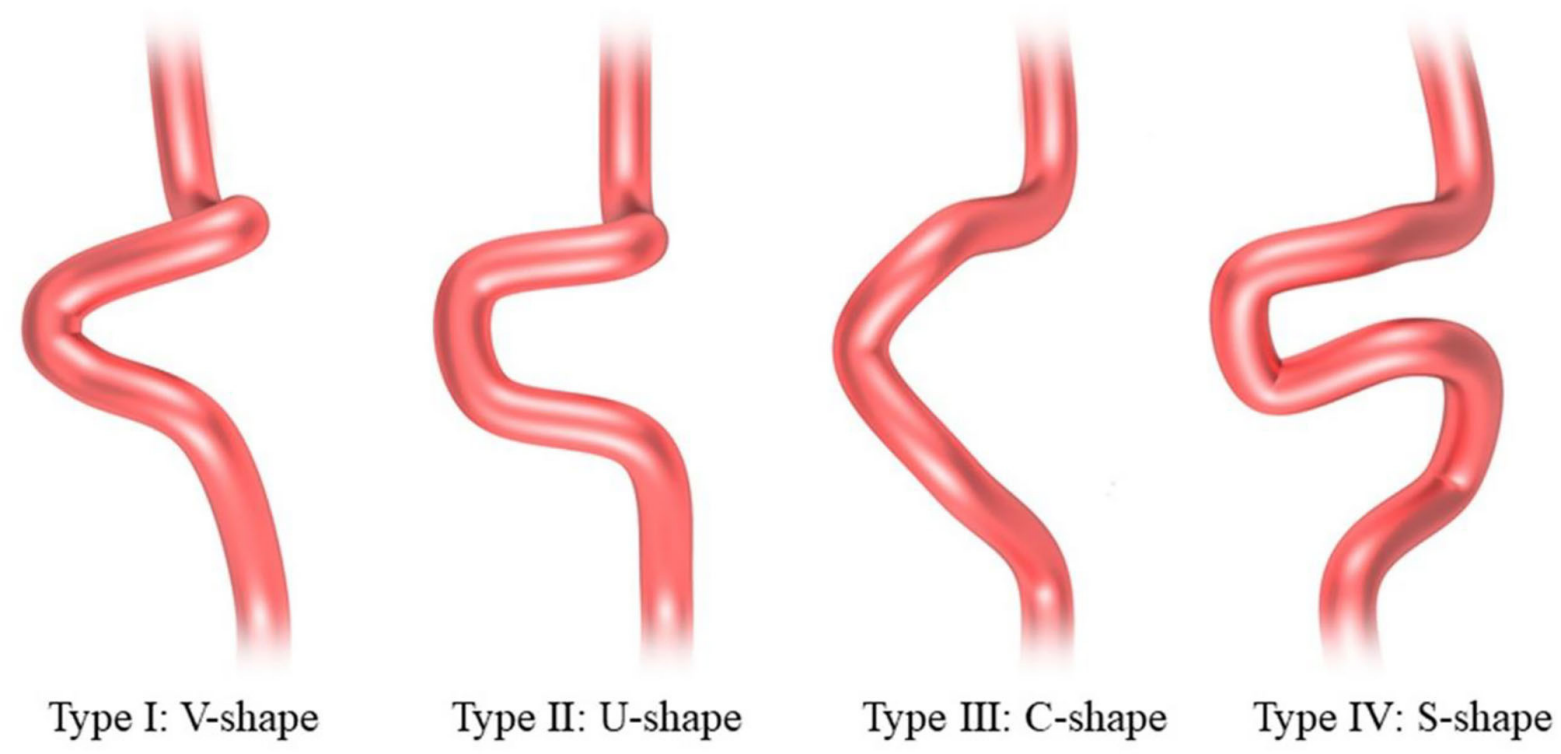
Examples of carotid siphon internal carotid artery (ICA) tortuosity.

**Figure 3:**
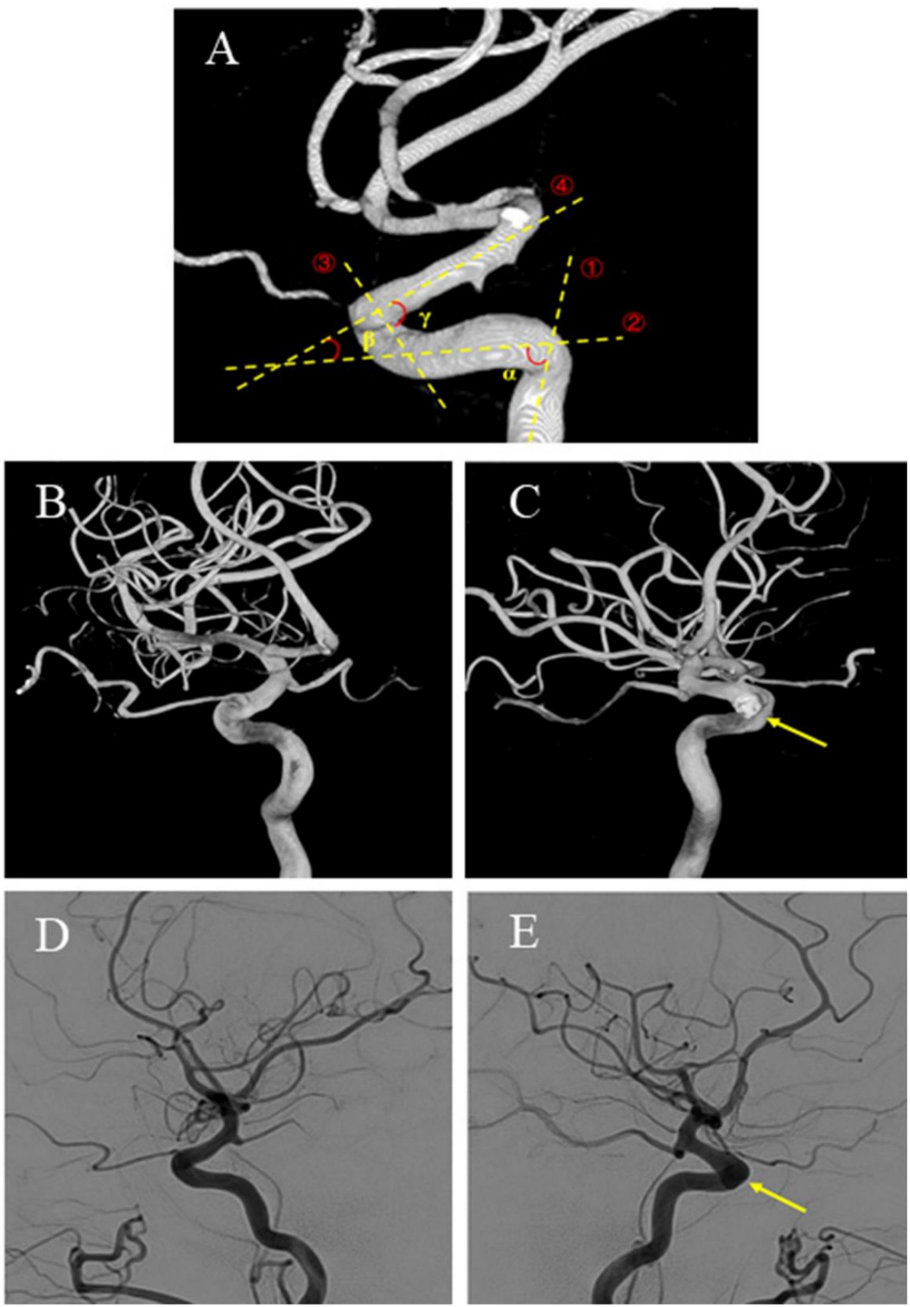
Angle measurement in a patient with unilateral carotid siphon aneurysms. **(A)**①, Passes through the centerline of the vertical cavernous segment vessel.②, Passes through the centerline of the horizontal cavernous segment vessel.③, Passes through the centerline of the clinoid segment vessel.④, Passes through the centerline of the ophthalmic segment vessel. α, Carotid siphon posterior bend angle. β, Carotid siphon anterior bend angle. γ, Carotid siphon Clinoid@Ophthalmic bend angle. Images **(B–E)** from the same patient. **(B,C)** Volume rendering image of bilateral three-dimensional rotational angiography data of a patient with unilateral paraclinoid aneurysm. **(C)** The aneurysmal side (yellow arrow). **(D)** DSA of the unaffected contralateral side. **(E)** DSA of the aneurysmal side (yellow arrow). 3D-DSA: three-dimensional digital subtraction angiography.

The 3D models of the parent vessels and aneurysms were tumbled freely and measured *via* our workplace system. The carotid siphons were morphologically classified into four types: “V” shaped, “U” shaped, “C” shaped, and “S” shaped as described by Zhang et al. (20) (Figure 2). Rotational angiography data sets were obtained from the post-processing workstation to reconstruct the 3D vessel, the carotid siphon surface, and the carotid siphon skeleton (centerline). The local vessel centerline guided multiplanar reconstruction into the volume at the site of the carotid siphon, ensuring that the plane was close to the center of the vessels. Carotid siphon angles were measured at the intersection of the two lines serving as midpoints of the arterial diameters of the segments under question (Figure 3) (21, 22). Angles were measured in the direction of flow to represent the deviation from the distal segment with respect to the reference proximal segment (angle deviation). The acquired measurements included: angle of the posterior knees of the carotid siphon (α), angle of the anterior knees of the carotid siphon (β), the angle between the clinoid segment and ophthalmic segment (γ). A total of two experienced neuroradiologists, assisted by a dedicated Philips engineer, independently analyzed 3D DSA images and made measurements for all patients. Intraclass correlation was used to assess the inter-rater reliability for morphology measurement, and it was found to be in good agreement between the two neuroradiologists (ICC = 0.874 95% 0.833–0.916) (23).

### Statistical Analyses

A total of three categories of carotid siphon angles were analyzed: (i) carotid siphon angles on the side of the carotid siphon aneurysm; (ii) unaffected contralateral side carotid siphon angles; (iii) control from patients with no aneurysm. Normally distributed data were expressed as mean ± SD. A Kolmogorov–Smirnov test was applied to determine the normality of the data and its distribution. Data were normally distributed and analyzed by *t*-test. Non-normal distributed data were analyzed by the Mann–Whitney U test and presented as the median and interquartile range (IQR). Qualitative parameters were compared by the chi-square test (χ2) and the Student's *t*-test was employed to examine the statistical differences between carotid siphon angles. We also assessed the angle of the aneurysm side and the contralateral side. An independent sample *t*-test was applied to analyze and compare data between patients with aneurysms and controls. A separate paired analysis was performed to compare the corresponding angle differences when data were available on both aneurysmal and normal contralateral carotid siphon angles, from the same patient. Comparison of carotid siphon angle parameters for the aneurysms at different segment locations was achieved by Student's *t*-test. Independent risk factors for paraclinoid aneurysms were analyzed using binary logistic regression. The area under the curve (AUC) and the optimal cut-off values for α, β, and γ angles were assessed by receiver operator characteristics (ROCs) curves. All the analyses were performed using SPSS statistics 24.0 (SPSS, version 24.0) and *P*-values < 0.05 denoted statistical significance.

## Results

### Patient Demographics

A total of 42 patients were diagnosed with symptomatic paraclinoid aneurysms with the control group comprising 42 healthy patients with no intracranial aneurysm. Data on both aneurysmal and normal contralateral carotid siphons were obtained from a subset of 31 patients. All the patients in the paraclinoid aneurysm group had a unilateral aneurysm (18 and 24 on the right and left, respectively). Patients with paraclinoid aneurysms had increased “U”-shaped and “V”-shaped carotid siphons and Table 1 summarizes the aneurysmal morphological parameters. The paraclinoid aneurysm group comprised 12 males and 30 females, aged 58.2 ± 10.1 (range 44–77) years. The control group comprised 12 males and 30 females, aged 56.1 ± 13.0 (range 37–79) years. The analysis demonstrated no significant differences between the two groups regarding parameters such as age, gender, hypertension, smoking habit, hyperlipidemia, and diabetes mellitus.

**Table 1:** Comparison of patients with paraclinoid aneurysms with the age- and sex-matched control group.

	**Patients (*n* = 42)**	**Controls (*n* = 42)**	***P*-value**
Age (Y)	58.2 ± 10.1	56.1 ± 13.0	0.538
Gender			
Male	12	12	1.0^a^
Female	30	30	
Hypertension	16	14	0.649^a^
Smoking	15	12	0.483^a^
Hyperlipidaemia	9	12	0.450^a^
Diabetes mellitus	11	8	0.434^a^
Siphon type, *n* (%)			
“U”	16 (38.1%)	17 (40.5%)	
“V”	15 (35.7%)	13 (31.0%)	
“C”	5 (11.9%)	8 (19.0%)	
“S”	6 (14.3%)	4 (9.5%)	
Aneurysm dome size	3.97 ± 1.12		
(mm, mean ± SD)			
Aneurysm neck size	3.21 ± 1.63		
(mm, mean ± SD)			

*SD, standard deviation.*

*a, For variable gender, the chi-square test (χ^2^) was performed*.

### Univariate Statistical Analysis

**Figure 4:**
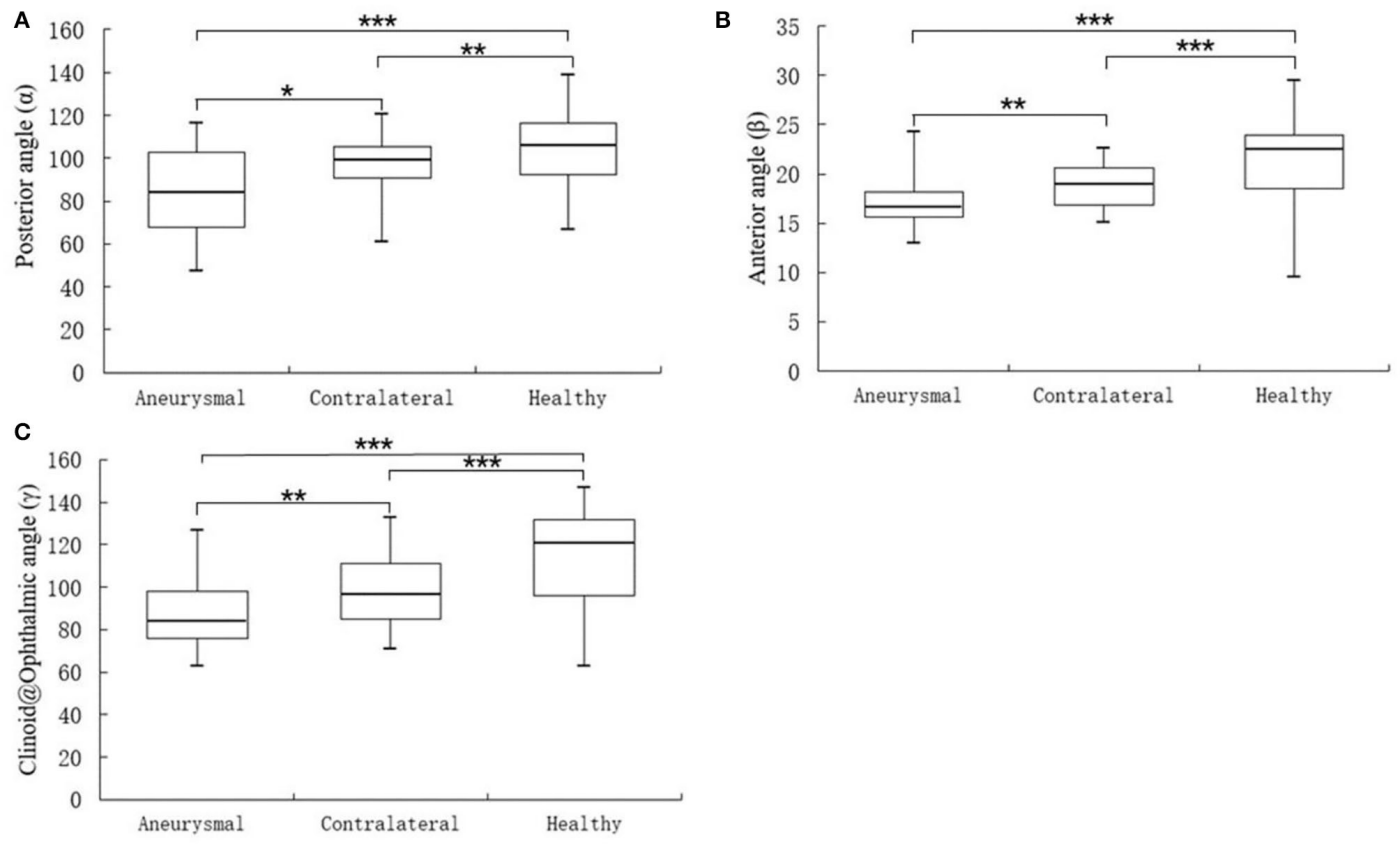
Statistical differences between aneurysmal, contralateral, and healthy carotid siphon angle for angles α **(A)**, β **(B)**, and γ **(C)**. ^*^P < 0.05, ^**^P < 0.01, ^***^P < 0.001.

The α, β, and γ angles were found to be significantly smaller in the aneurysmal carotid siphon when compared with the carotid siphon with no aneurysm involvement (Figure 4). Patients with paraclinoid aneurysms exhibited a significantly smaller posterior angle [median (IQR), 85.55° (67.85–102.90°)] when compared with the unaffected contralateral group [median (IQR), 97.50° (90.70–105.50°), *P* < 0.05] and control patients with no aneurysm [median (IQR), 103.55° (92.55–116.50°), *P* < 0.001] (Table 2). Similarly, the non-aneurysmal contralateral group exhibited a significantly smaller posterior angle when compared with control patients with no aneurysms (*P* < 0.01).

**Table 2:** Univariate statistical analysis of angles α, β, and γ.

	**Aneurysmal (A)**	**Contralateral (C)**	**Healthy (H)**
Posterior angle (α)	85.55° (67.85–102.90°)	97.50° (90.70–105.50°)	103.55° (92.55–116.50°)
	A vs C *P* < 0.05	C vs H *P* < 0.01	A vs H *P* < 0.001
Anterior angle (β)	16.95° (15.63–18.20°)	19.30° (16.90–20.60°)	21.35° (18.48–23.98°)
	A vs C *P* < 0.01	C vs H *P* < 0.001	A vs H *P* < 0.001
Clinoid@Ophthalmic angle (γ)	81.30° (75.83–98.30°)	95.40° (85.00–111.00°)	115.35° (96.08–131.78°)
	A vs C *P* < 0.01	C vs H *P* < 0.001	A vs H *P* < 0.001

*Shown are the median and interquartile range for patients with paraclinoid aneurysms, unaffected contralateral groups, and healthy control groups*.

A comparison of the anterior angle between groups revealed significantly smaller β angle in patients with paraclinoid aneurysms [median (IQR), 16.95° (15.63–18.20°)] when compared to patients with unaffected sides [median (IQR), 19.30°(16.90–20.60°), *P* < 0.01] and control patients with no aneurysms [median (IQR), 21.35° (18.48–23.98°), *P* < 0.001]. In addition, the smaller β angle in the unaffected contralateral group was statistically significant when compared to that of healthy subjects (*P* < 0.001).

By comparing the Clinoid@Ophthalmic angle, we found that the γ angle was significantly smaller in patients with paraclinoid aneurysms [median (IQR), 81.30° (75.83–98.30°)] when compared with subjects with unaffected sides [median (IQR), 95.40°(85.00–111.00°), *P* < 0.01] and control patients with no aneurysms [median (IQR), 115.35° (96.08–131.78°), *P* < 0.001]. Finally, the γ angle in the unaffected contralateral group was significantly smaller when compared to that of healthy subjects (*P* < 0.001).

The mean aneurysm sizes and the mean neck sizes were 3.97 ± 1.12 mm, and 3.21±1.63 mm, respectively. No correlation between α, β, and γ angles and aneurysm size of aneurysm neck size was reported. Notably, the inclination carotid siphon angles were not statistically significant for aneurysm morphology.

### Optimal Cutoff Thresholds

**Figure 5:**
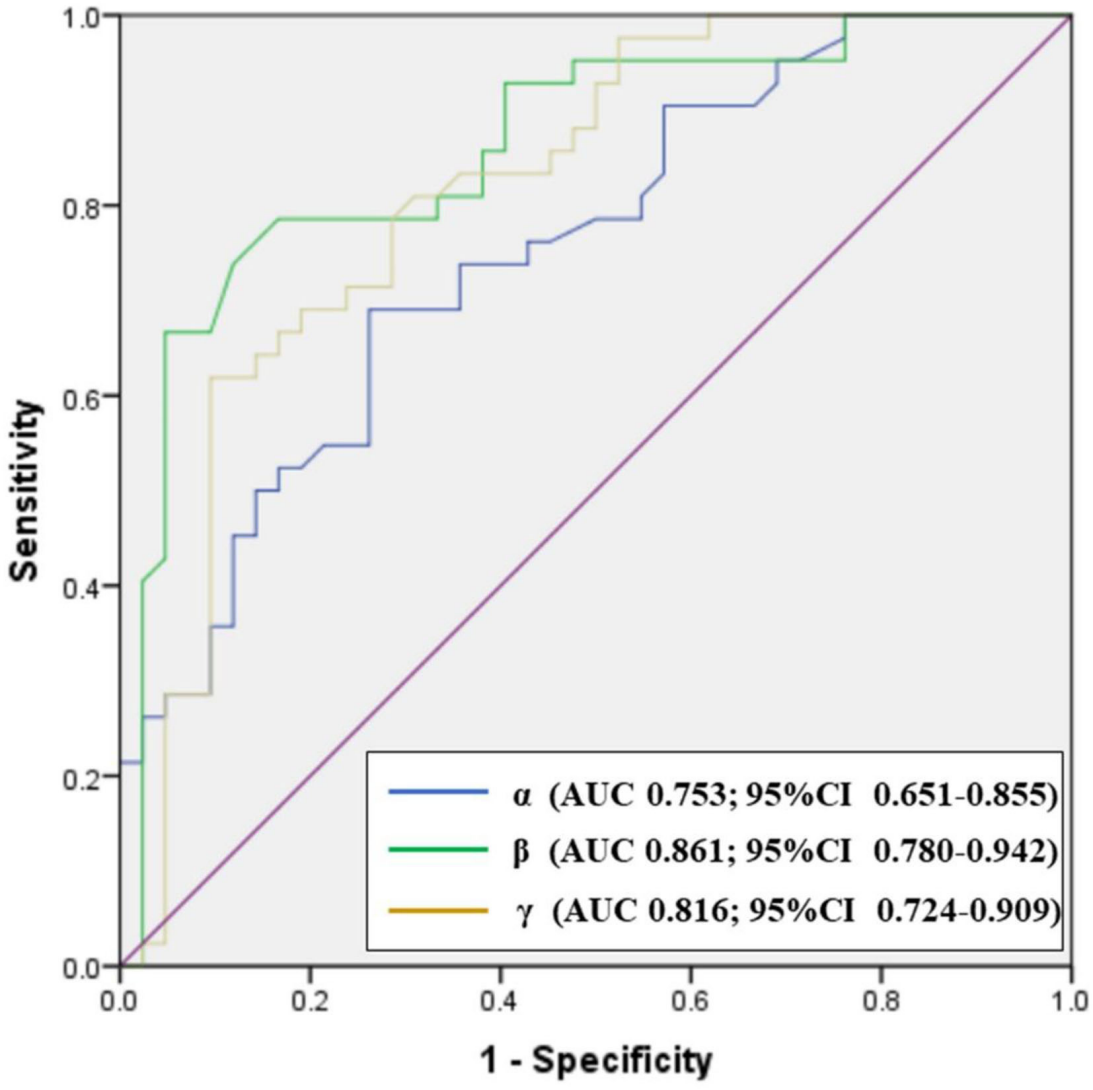
Receiver operator characteristics (ROC) plot showing the performance of carotid siphon angles in discriminating between paraclinoid aneurysms groups and healthy control groups. CI: confidence interval.

Analysis of ROCs revealed optimal cutoff thresholds for the carotid siphon angles and distinguished between patients with paraclinoid aneurysms and healthy controls (Figure 5). Results showed that the anterior angle (β) was the best identifier in discriminating between aneurysmal and non-aneurysmal carotid siphons, with an optimal β threshold of 18.25° (AUC, 0.861), a sensitivity of 78.6%, and a specificity of 83.3%. The optimal posterior angle (α) threshold was 98.55° (AUC, 0.753), with a sensitivity and specificity of 69.0 and 73.8%, respectively. The optimal Clinoid@Ophthalmic angle (γ) threshold was 85.3° (AUC, 0.816), with a sensitivity and specificity of 61.9% and 90.5%, respectively.

### Matched Pair Analysis

Bilateral DSA images were available for 31 patients with unilateral paraclinoid aneurysms. Aneurysmal carotid siphon angles exhibited a significantly different carotid siphon angle when compared to their unaffected contralateral side (Table 3). This was true for posterior angle [median [IQR], 78.30° (65.70–94.70°) aneurysmal vs. median (IQR), 97.50° (90.70–105.50°) contralateral, *P* < 0.001], for anterior angle [median (IQR), 16.10° (15.00–18.00°) aneurysmal vs. median (IQR), 19.30° (16.90–20.60°) contralateral, *P* < 0.001], and Clinoid@Ophthalmic angle [median (IQR), 78.50° (71.20–87.30°) aneurysmal vs. median (IQR), 95.40° (85.00–111.00°) contralateral, *P* < 0.001].

**Table 3:** Matched pair statistical analysis of aneurysmal and non-aneurysmal contralateral for α, β, and γ angles within same patients.

	**Aneurysmal (A) *n* = 31**	**Contralateral (C) *n* = 31**	***P*-value**
Posterior angle (α)	78.30° (65.70–94.70°)	97.50° (90.70–105.50°)	*P* < 0.001
Anterior angle (β)	16.10° (15.00–18.00°)	19.30° (16.90–20.60°)	*P* < 0.001
Clinoidal@Ophthalmic angle (γ)	78.50° (71.20–87.30°)	95.40° (85.00–111.00°)	*P* < 0.001

*Values shown are medians (interquartile ranges)*.

### Aneurysm Location Analysis

A total of two categories of aneurysm location were used, including 17 (39%) that originated away from the clinoid segment and 25 (61%) away from the ophthalmic segment, and subgroup analysis yielded the following results: (i) matched comparison of α, β, and γ angles based on aneurysm location showed a significant association between aneurysm location and smaller flow angle (Table 4); (ii) the anterior angle was significantly higher in 25 patients with the ophthalmic segment when compared to those patients with the clinoid segment [median (IQR), 17.40°(16.35–20.00°) vs 15.70°(14.85–17.30°), *P* < 0.01] but the Clinoid@Ophthalmic angle was significantly higher in 17 patients with the clinoid segment [median (IQR), 78.50°(71.65–87.95°) vs. 98.20° (78.95–116.90°), *P* < 0.01]; (iii) the posterior angle showed no significant differences between patients with an ophthalmic segment and those with a clinoid segment [median (IQR), 94.50° (67.70–102.30°) vs. 81.00° (71.75–103.90°), *P* = 0.749] (Table 4).

**Table 4:** A comparison of the carotid siphon angle morphology of aneurysms originating from between the ophthalmic segment (C6) and clinoid segment (C5).

	**Ophthalmic segment group *N* = 25**	**Clinoid segment group *N* = 17**	
Posterior angle (α)	94.50° (67.70–102.30°)	81.00° (71.75–103.90°)	*P* = 0.749
Anterior angle (β)	17.40° (16.35–20.00°)	15.70° (14.85–17.30°)	*P* < 0.01
Clinoidal@Ophthalmic angle (γ)	78.50° (71.65–87.95°)	98.20° (78.95–116.90°)	*P* < 0.01

*Values shown are medians (interquartile ranges)*.

### Multivariate Analysis

After adjusting for confounding factors such as hypertension, smoking, hyperlipidemia, and diabetes mellitus, our analysis showed that the posterior angle (α) (OR = 0.953, 95% CI: 0.914–0.993, *P* < 0.05), anterior angle(β) (OR = 0.690, 95% CI: 0.549–0.867, *P* < 0.001), and Clinoid@Ophthalmic angle(γ) (OR = 0.958, 95% CI: 0.926–0.991, *P* < 0.01) were independently associated with the presence of paraclinoid aneurysms (Table 5).

**Table 5:** Independent factors associated with paraclinoid aneurysms group.

			**95% CI**	
	***p*-value**	**OR**	**Lower**	**Upper**
Hypertension	0.854	1.134	0.297	4.330
Smoking	0.225	2.350	0.591	9.339
Hyperlipidaemia	0.115	0.246	0.043	1.405
Diabetes mellitus	0.531	1.656	0.342	8.029
Posterior angle (α)	<0.05	0.953	0.914	0.993
Anterior angle (β)	<0.001	0.690	0.549	0.867
Clinoid@Ophthalmic angle (γ)	<0.01	0.958	0.926	0.991

*OR: odds ratio; CI: confidence interval*.

## Discussion

Previous studies have investigated a possible correlation between adjacent vessels and the presence of aneurysms at the internal carotid artery (10, 24, 25). Adel et al. (10) evaluated the mean and peak curvatures of the intracranial ICA and showed that the presence of sidewall aneurysms was highly correlated with curvature in all the genders. Non-aneurysmal ICAs were more curved in women than men, which could explain the increased predisposition to aneurysm formation in women. Similarly, Hu et al. (24) reported that the correlation between the angles at C7 and C6, the ICA, and the presence of symptomatic PcoA; with smaller angles being significantly associated with increased frequency of PcoA. Finally, Rosato et al. (25) showed that ICA angulation at the PcoA origin is significantly higher in vessels harboring PcoA aneurysms when compared with non-aneurysmal ICAs. Furthermore, the carotid siphon constitutes an exceptional zone because of the conjunction of a tortuous vessel segment and high blood flow. Evidence from previous studies has indicated that the two distinct bends of the carotid siphon are defined as anterior angle and posterior angle, connected by a horizontal section (26, 27). Lin et al. (27), using 3D images from 83 patients with intracranial ICA aneurysms, proposed a classification for the carotid siphon based on measurements of the anterior and posterior angle geometry, and used this for interventional treatment therapy in preoperative decision making.

Unlike earlier reports, here we incorporated not only data derived from the carotid siphon of the posterior and anterior angles but also measured the angle of C5 and C6 (Clinoid@Ophthalmic) to describe the carotid siphon. The Clinoid@Ophthalmic angle not only represents the turning point of the internal carotid artery but also the opening of the ophthalmic artery. The findings of Liu et al. (18) who analyzed morphological parameters from a group with ruptured or unruptured paraclinoid aneurysms, suggesting that the association of irregular shape, larger energy loss, and concentrated inflow jet are associated with aneurysm rupture.

In this study, due to clinical practicality, we used a simple and reproducible method and selected only three basic geometrical parameters as independent variables, while other complicated geometrical and three-dimensional parameters, such as non-sphericity index, inflow angle, and undulation index, were excluded. We focused only on carotid siphon angles to eliminate variables with parent arteries, associated with a refined statistical analysis to identify independent variables caused by angle variation. Our results showed significant differences in the carotid siphon angle between patients who were aneurysmal and controls, particularly in the subgroup analyses. Further analysis demonstrated that the anterior angle (AUC, 0.861) was potentially a more sensitive indicator of increased risk of paraclinoid aneurysm. Multivariate analysis demonstrated an independent association of the posterior angle, anterior angle, and Clinoid@Ophthalmic angle with the presence of symptomatic paraclinoid aneurysms. Evidence suggests that the angles of the winding part may be associated with shear stress on the artery wall and the mechanism of naturally occurring cerebral aneurysm because the pressure in these parts was higher than juxtaposed arteries. It has been speculated that the bending of the carotid siphon induces a greater impact force of turbulent flow against the artery walls and causes changes in wall shear stress (WSS) and the wall shear stress gradient (WSSG), which can alter endothelial cell function thereby promoting arterial remodeling. We indeed found a significant increase in the carotid siphon tortuosity in the aneurysm group. In addition, when compared with extracranial arteries, intracranial arteries lack an external elastic layer, making them susceptible to the aneurysms.

To minimize potential bias from different locations, we focused on single paraclinoid aneurysms in different segments. A novel finding in this study was that while clinoid and ophthalmic segment aneurysms are sidewall aneurysms, the carotid siphon showed significantly different morphological aspects. Of note, when the aneurysm is in the clinoid segment, the anterior angle involved is narrower when compared to that seen in the ophthalmic segment. In contrast, when the aneurysm is located in the ophthalmic segment, the Clinoid@Ophthalmic angle involved is narrower when compared to that located in the clinoid segment. Clinoid segment aneurysms are anatomically adjacent to the anterior angle, and most of the ophthalmic segment aneurysms are anatomically adjacent to the Clinoid@Ophthalmic angle. We assumed that such an angle could lead to aneurysm formation, because it would predispose blood flow to deviation and impact on the blood vessel wall, leading to changes in WSS.

Evidence from compelling neuroimaging results suggests a relationship between increased carotid siphon tortuosity and paraclinoid aneurysms. Sugiyama et al. (28) compared the carotid siphon to a meandering river in the natural world and reported that meandering rivers experience less erosion on the side with the lesser curve when compared with the side with the greater curve. Also, the riverbed is more easily formed on the side with the lesser curve and it experiences a lower flow velocity. Anatomically, vessels with more curvature are associated with lower WSS and higher wall shear oscillations. The flow of blood into the torturous part of the blood vessel changes the pattern of blood flow from laminar to turbulent flow, producing areas of locally lower WSS on the inner wall of the bend and areas of higher WSS at the outer wall of the bend (29, 30). According to the high-flow theory, the abnormal increase in WSS stretches collagen and elastic fibers causing focal endothelial injury and structural changes. These events cause a gradual dilatation and enlargement of the intracranial aneurysm wall. However, the low-WSS theory states that the blood flow in the lumen of the artery slows down, and thus recruits inflammatory cells such as macrophages, lymphocytes, neutrophils, and platelets. Further investigation has revealed that the release of pro-inflammatory mediators and oxidative stress promotes vascular injury and endothelial dysfunction. An injury to endothelial cells inflicted by different stressors induces matrix protein degradation, the production of vasoconstrictive agents and oxidants, leukocyte rolling and adhesion, integrin-mediated firm adhesion, and diapedesis, all resulting in arterial vessel wall remodeling (31). Thus, assessment of tortuosity with such deviant morphology could potentially enable follow-up for assessment of longitudinal risk evaluation of aneurysm initiation. Further validation and in-depth exploration of this hypothesis require computational fluid dynamic studies, which will be the focus of future research. Future prospective studies are needed to elucidate potential causal vs. associative relationships.

This study has established evidence and suggests a correlation between aneurysm occurrence and vessel angulation but does not demonstrate causality. It is expected that the presence of an aneurysm could have changed the morphology of the vessel and increased the bending of the carotid siphon at the ICA. However, our analysis showed no association between carotid siphon angles and aneurysm sizes. It is notable that unaffected contralateral groups had significantly smaller carotid siphon angles when compared to healthy controls, demonstrating that increased vessel tortuosity may precede aneurysm formation. This is consistent with the results of previous studies (14, 32). Tutino et al. used rabbit models to show that increased vascular tortuosity preceded the establishment of aneurysms after bilateral carotid ligation (29). Kornelia et al. also compared ICA tortuosity to age and gender-matched healthy controls, as well as objectively assessing ICA tortuosity using five mathematical parameters based on angle and length. They discovered that aneurysm and SAH patients had increased tortuosity and a smaller mean ICA diameter than healthy controls, and concluded that the presence of aneurysms did not significantly affect the tortuosity of their supplying arteries (14).

### Limitations

There were some limitations to this study. First, we focused uniquely on anatomical factors but other factors, such as clinical history and genetic factors, are also considered to have a vital role in the natural history of aneurysms and it will be necessary to add these risk factors to future studies. Second, as a two-center and retrospective study, potential regional and selection biases may have existed in the patient population and therefore, a more prospective multi-center, large sample study is warranted. Third, this study was based on cross-sectional data, and as such, future longitudinal studies analyzing larger sample sizes are needed to verify the reproducibility of the results. Fifth, owing to the aforementioned limitations, it remains poorly understood whether the presence of aneurysms affects the geometry of the parent vessels and prospective studies would be needed to further address this issue.

## Conclusions

The morphology of the carotid siphon at the site of paraclinoid aneurysms is characterized by a significantly sharper bend in patients with paraclinoid aneurysms when compared with the contralateral or healthy carotid siphons. An optimal anterior angle threshold of 18.25° (78.6% specificity and 83.3% sensitivity) was established for the discrimination of patients with aneurysms from healthy controls. In addition, our data indicated that the posterior, anterior, and Clinoid@Ophthalmic angles of the carotid siphons might play an important role in the formation of paraclinoid aneurysms. These practical morphological parameters specific to paraclinoid aneurysms are easy to assess and may aid in risk assessment in these patients.

## Data Availability Statement

The original contributions presented in the study are included in the article/supplementary material, further inquiries can be directed to the corresponding authors.

## Ethics Statement

The studies involving human participants were reviewed and approved by the Ethics Committee of the Fourth Affiliated Hospital of Anhui Medical University. Written informed consent for participation was not required for this study in accordance with the national legislation and the institutional requirements.

## Author Contributions

SL analyzed the data, framed it, and wrote the manuscript. YJ analyzed data and edited manuscripts. XZ and XW collected the imaging data. YZ, LJ, and GL collected the clinical data. TJ and XZ designed and supervised the paper and finalized the manuscript. All the authors reviewed the manuscript and made a significant contributions to this manuscript. All authors contributed to the article and approved the submitted version.

## Funding

This work was supported by the Research Fund of Anhui Institute of translational medicine (grant number 2021zhyx-C72), the National Key R & D Program (grant number 2021YFE0100100), and the Intra-hospital Fund of the Fourth Affiliated Hospital of Anhui Medical University (grant number 2021YKJ009).

## Conflict of Interest

XZ is employed by the Philips (China) Investment Company, Shanghai, China. The remaining authors declare that the research was conducted in the absence of any commercial or financial relationships that could be construed as a potential conflict of interest.

## Publisher's Note

All claims expressed in this article are solely those of the authors and do not necessarily represent those of their affiliated organizations, or those of the publisher, the editors and the reviewers. Any product that may be evaluated in this article, or claim that may be made by its manufacturer, is not guaranteed or endorsed by the publisher.

## References

[1] Ou C Chong W Duan CZ Zhang X Morgan M Qian Y. preliminary investigation of radiomics differences between ruptured and unruptured intracranial aneurysms. Eur Radiol. (2021) 31:2716–25. 10.1007/s00330-020-07325-3 33052466

[2] Xiang J Natarajan SK Tremmel M Ma D Mocco J Hopkins LN. Hemodynamic-morphologic discriminants for intracranial aneurysm rupture. Stroke. (2011) 42:144–52. 10.1161/STROKEAHA.110.592923 21106956 PMC3021316

[3] Piccinelli M Bacigaluppi S Boccardi E Ene-Iordache B Remuzzi A Veneziani A. Geometry of the internal carotid artery and recurrent patterns in location, orientation, and rupture status of lateral aneurysms: an image-based computational study. Neurosurgery. (2011) 68:1270–85. 10.1227/NEU.0b013e31820b5242 21273931

[4] Raghavan ML Ma B Harbaugh RE. Quantified aneurysm shape and rupture risk. J Neurosurg. (2005) 102:355–62. 10.3171/jns.2005.102.2.0355 15739566

[5] Matsukawa H Fujii M Akaike G Uemura A Takahashi O Niimi Y. Morphological and clinical risk factors for posterior communicating artery aneurysm rupture. J Neurosurg. (2014) 120:104–10. 10.3171/2013.9.JNS13921 24160476

[6] Ou C Liu J Qian Y Chong W Zhang X Liu W. Rupture risk assessment for cerebral aneurysm using interpretable machine learning on multidimensional data. Front Neurol. (2020) 11:570181. 10.3389/fneur.2020.570181 33424738 PMC7785850

[7] Sforza DM Putman CM Cebral JR. Hemodynamics of cerebral aneurysms. Annu Rev Fluid Mech. (2009) 41:91–107. 10.1146/annurev.fluid.40.111406.102126 19784385 PMC2750901

[8] Sharifi A Niazmand H. Analysis of flow and LDL concentration polarization in siphon of internal carotid artery: non-newtonian effects. Comput Biol Med. (2015) 65:93–102. 10.1016/j.compbiomed.2015.08.002 26313530

[9] Valen-Sendstad K Piccinelli M Steinman DA. High-resolution computational fluid dynamics detects flow instabilities in the carotid siphon: implications for aneurysm initiation and rupture? J Biomech. (2014) 47:3210–6. 10.1016/j.jbiomech.2014.04.018 25062933

[10] Lauric A Safain MG Hippelheuser J Malek AM. High curvature of the internal carotid artery is associated with the presence of intracranial aneurysms. J Neurointerv Surg. (2014) 6:733–9. 10.1136/neurintsurg-2013-010987 24335804

[11] Roy D Raymond J Bouthillier A Bojanowski MW Moumdjian R. L'Espérance G. Endovascular treatment of ophthalmic segment aneurysms with Guglielmi detachable coils. AJNR Am J Neuroradiol. (1997) 18:1207–15. 9282843 PMC8338007

[12] Hoh BL Carter BS Budzik RF Putman CM Ogilvy CS. Results after surgical and endovascular treatment of paraclinoid aneurysms by a combined neurovascular team. Neurosurgery. (2001) 48:78–90. 10.1227/00006123-200101000-00014 11152364

[13] Bae DH Kim JM Won YD Choi KS Cheong JH Yi HJ Kim CH. Clinical outcome of paraclinoid internal carotid artery aneurysms after microsurgical neck clipping in comparison with endovascular embolization. J Cerebrovasc Endovasc Neurosurg. (2014) 16:225–34. 10.7461/jcen.2014.16.3.225 25340024 PMC4205248

[14] Kliś KM Krzyzewski RM Kwinta BM Stachura K Gasowski J. Tortuosity of the internal carotid artery and its clinical significance in the development of aneurysms. J Clin Med. (2019) 8:237. 10.3390/jcm8020237 30759737 PMC6406528

[15] Waihrich E Clavel P Mendes G Iosif C Kessler IM Mounayer C. Influence of anatomic changes on the outcomes of carotid siphon aneurysms after deployment of flow-diverter stents. Neurosurgery. (2018) 83:1226–33. 10.1093/neuros/nyx618 29444328

[16] Lauric A Hippelheuser J Safain MG Malek AM. Curvature effect on hemodynamic conditions at the inner bend of the carotid siphon and its relation to aneurysm formation. J Biomech. (2014) 47:3018–27. 10.1016/j.jbiomech.2014.06.042 25062932 PMC4163091

[17] Ou C Li C Qian Y Duan CZ Si W Zhang X. Morphology-aware multi-source fusion-based intracranial aneurysms rupture prediction. Eur Radiol. (2022) 10.1007/s00330-022-08608-7. [Epub ahead of print]. 35182202

[18] Liu J Xiang J Zhang Y Wang Y Li H Meng H. Morphologic and hemodynamic analysis of paraclinoid aneurysms: ruptured versus unruptured. J Neurointerv Surg. (2014) 6:658–63. 10.1136/neurintsurg-2013-010946 24220206

[19] Bouthillier A van Loveren HR Keller JT. Segments of the internal carotid artery: a new classification. Neurosurgery. (1996) 38:425–33. 10.1227/00006123-199603000-00001 8837792

[20] Zhang C Pu F Li S Xie S Fan Y Li D. Geometric classification of the carotid siphon: association between geometry and stenoses. Surg Radiol Anat. (2013) 35:385–94. 10.1007/s00276-012-1042-8 23183849

[21] Silva Neto ÂR Câmara RL Valença MM. Carotid siphon geometry and variants of the circle of willis in the origin of carotid aneurysms. Arq Neuropsiquiatr. (2012) 70:917–21. 10.1590/S0004-282X2012001200003 23295418

[22] Waihrich E Clavel P Mendes GAC Iosif C Moraes Kessler I Mounayer C. Influence of carotid siphon anatomy on brain aneurysm presentation. AJNR Am J Neuroradiol. (2017) 38:1771–5. 10.3174/ajnr.A5285 28684458 PMC7963716

[23] Koo TK Li MY A. Guideline of selecting and reporting intraclass correlation coefficients for reliability research. Med J Chiropr Med. (2016) 15:155–63. 10.1016/j.jcm.2016.02.012 27330520 PMC4913118

[24] Hu T Wang D. Association between anatomical variations of the posterior communicating artery and the presence of aneurysms. Neurol Res. (2016) 38:981–7. 10.1080/01616412.2016.1238662 27731782

[25] Rosato R Comptdaer G Mulligan R Breton JM Lesha E Lauric A. Increased focal internal carotid artery angulation in patients with posterior communicating artery aneurysms. J Neurointerv Surg. (2020) 12:1142–7. 10.1136/neurintsurg-2020-015883 32447300

[26] Harrigan MR Deveikis JP Handbook of Cerebrovascular Disease and Neurointerventional Technique. 2nd ed. Dordecht: Humana Press (2013).

[27] Lin LM Colby GP Jiang B Uwandu C Huang J Tamargo RJ. Classification of cavernous internal carotid artery tortuosity: a predictor of procedural complexity in Pipeline embolization. J Neurointerv Surg. (2015) 7:628–33. 10.1136/neurintsurg-2014-011298 24996435

[28] Sugiyama N Fujii T Yatomi K Teranishi K Oishi H Arai H. Endovascular treatment for lateral wall paraclinoid aneurysms and the influence of internal carotid artery angle. Neurol Med Chir (Tokyo). (2021) 61:275–83. 10.2176/nmc.oa.2020-0307 33716235 PMC8048120

[29] Zhang C Xie S Li S Pu F Deng X Fan Y. Flow patterns and wall shear stress distribution in human internal carotid arteries: the geometric effect on the risk for stenoses. J Biomech. (2012) 45:83–9. 10.1016/j.jbiomech.2011.10.001 22079384

[30] Foutrakis GN Yonas H Sclabassi RJ. Saccular aneurysm formation in curved and bifurcating arteries. AJNR Am J Neuroradiol. (1999) 20:1309–17. 10472991 PMC7055997

[31] Giotta Lucifero A Baldoncini M Bruno N Galzio R Hernesniemi J Luzzi S. Shedding the light on the natural history of intracranial aneurysms: an updated overview. Medicina (Kaunas). (2021) 57:742. 10.3390/medicina57080742 34440948 PMC8400479

[32] Tutino VM Mandelbaum M Choi H Pope LC Siddiqui A Kolega J. Aneurysmal remodeling in the circle of willis after carotid occlusion in an experimental model. J Cereb Blood Flow Metab. (2014) 34:415–24. 10.1038/jcbfm.2013.209 24326393 PMC3948116

